# Differential vulnerability of the dentate gyrus to tauopathies in dementias

**DOI:** 10.1186/s40478-022-01485-7

**Published:** 2023-01-03

**Authors:** Allegra Kawles, Grace Minogue, Antonia Zouridakis, Rachel Keszycki, Nathan Gill, Caren Nassif, Christina Coventry, Hui Zhang, Emily Rogalski, Margaret E. Flanagan, Rudolph Castellani, Eileen H. Bigio, M. Marsel Mesulam, Changiz Geula, Tamar Gefen

**Affiliations:** 1 Mesulam Center for Cognitive Neurology & Alzheimer’s Disease, Northwestern University Feinberg School of Medicine, 300 E. Superior Street, Tarry Building, 8th Floor, Chicago, IL 60611 USA; 2 Department of Psychiatry & Behavioral Sciences, Northwestern University Feinberg School of Medicine, Chicago, IL USA; 3 Department of Preventive Medicine, Northwestern University Feinberg School of Medicine, Chicago, IL USA; 4 Department of Pathology, Northwestern University Feinberg School of Medicine, Chicago, IL USA; 5 Department of Neurology, Northwestern University Feinberg School of Medicine, Chicago, IL USA; 6 Department of Cell and Developmental Biology, Northwestern University Feinberg School of Medicine, Chicago, USA

**Keywords:** Dentate gyrus, Frontotemporal lobar degeneration, Tauopathy, Primary progressive aphasia

## Abstract

The dentate gyrus (DG), a key hippocampal subregion in memory processing, generally resists phosphorylated tau accumulation in the amnestic dementia of the Alzheimer’s type due to Alzheimer’s disease (DAT-AD), but less is known about the susceptibility of the DG to other tauopathies. Here, we report stereologic densities of total DG neurons and tau inclusions in thirty-two brains of human participants with autopsy-confirmed tauopathies with distinct isoform profiles—3R Pick’s disease (PiD, N = 8), 4R corticobasal degeneration (CBD, N = 8), 4R progressive supranuclear palsy (PSP, N = 8), and 3/4R AD (N = 8). All participants were diagnosed during life with primary progressive aphasia (PPA), an aphasic clinical dementia syndrome characterized by progressive deterioration of language abilities with spared non-language cognitive abilities in early stages, except for five patients with DAT-AD as a comparison group. 51% of total participants were female. All specimens were stained immunohistochemically with AT8 to visualize tau pathology, and PPA cases were stained for Nissl substance to visualize neurons. Unbiased stereological analysis was performed in granule and hilar DG cells, and inclusion-to-neuron ratios were calculated. In the PPA group, PiD had highest mean total (granule + hilar) densities of DG tau pathology (*p* < 0.001), followed by CBD, AD, then PSP. PPA-AD cases showed more inclusions in hilar cells compared to granule cells, while the opposite was true in PiD and CBD. Inclusion-to-neuron ratios revealed, on average, 33% of all DG neurons in PiD cases contained a tau inclusion, compared to ~ 7% in CBD, 2% in AD, and 0.4% in PSP. There was no significant difference between DAT-AD and PPA-AD pathologic tau burden, suggesting that differences in DG burden are not specific to clinical phenotype. We conclude that the DG is differentially vulnerable to pathologic tau accumulation, raising intriguing questions about the structural integrity and functional significance of hippocampal circuits in neurodegenerative dementias.

**Supplementary Information:**

The online version contains supplementary material available at 10.1186/s40478-022-01485-7.

## Introduction

Neurodegenerative dementia syndromes are characterized by specific patterns of progressive cognitive and functional decline. Dementia of the Alzheimer’s type (DAT), characterized by initial presentation of memory impairments, is the most common. Memory impairments are thought to emerge because the hippocampus, a brain structure crucial for memory formation, is among the first regions to be affected by Alzheimer’s Disease (AD) neuropathology, the hallmarks of which are tau-containing neurofibrillary tangles (NFTs) and amyloid plaques. Hippocampal subregions are responsible for distinct functions in the formation of memory and learning [1, 2] and show variable vulnerability to AD pathology [3–5]. The dentate gyrus (DG) of the hippocampus is characterized by a single layer of densely packed granule cells, followed by an underlying polymorphic layer of long-projecting neurons in the hilus. The granule cells receive input from the entorhinal cortex and send excitatory output to hippocampal subfield Cornus Ammonis field 3 (CA3) via their mossy fibers, making the DG a key region in encoding of memories [6, 7]. Prior studies have demonstrated that the DG, particularly the granule cells, generally resists the formation of AD pathology in the typical amnestic dementia syndrome [8]. This finding is particularly puzzling as the DG plays a central role in associative memory and overall hippocampal neurocircuitry.

Not all neurodegenerative dementia syndromes affect memory systems at initial onset. For example, primary progressive aphasia (PPA) is a dementia syndrome characterized by progressive language deficits, with relative sparing of non-language functions, including memory, within approximately the first two years of clinical disease [9]. PPA is associated with distinct neuropathologies, with ~ 60% of cases showing features of frontotemporal lobar degeneration (FTLD) and ~ 40% showing AD pathology [10]. Regardless of pathologic diagnosis, all PPA cases share an aphasic phenotype due to focal atrophy in the language-dominant hemisphere corresponding to degeneration of the language network [11–15]. Our prior work demonstrated that the DAT-AD clinicopathologic phenotype is associated with higher NFT burden in the memory-associated entorhinal cortex, whereas the AD associated with PPA (PPA-AD) has an atypical distribution that favors the neocortical language network [16, 17]. These studies were among the first of many [10, 18–22] to establish clinicopathologic concordance between distribution of pathologic inclusions, anatomic location, and the clinical symptoms manifested during life.

In addition to AD pathology, the PPA syndrome can be due to other tauopathies of the frontotemporal lobar degeneration form (known as FTLD-tau), characterized by toxic intracellular aggregates of hyperphosphorylated tau that emerge in frontal and temporal regions [23]. Tau can be expressed in six different isoforms, with different amounts of N-terminal inserts and either 3 or four repeats in the microtubule binding domain (3R or 4R) [24]. Pathologic tau inclusions can be composed of either 3R tau (Pick disease; PiD) or 4R tau (corticobasal degeneration; CBD, or progressive supranuclear palsy; PSP), or both 3R and 4R tau (AD) [23, 25].

When viewed microscopically, brains of PPA patients with postmortem diagnoses of PiD have substantial tau pathology in the hippocampal complex, and in particular, the DG [26, 27]. Given the relative sparing of memory functions in PPA [28], the apparent susceptibility of the DG to tau inclusions in non-amnestic dementia raises interesting questions about the selective vulnerability of hippocampal neurons to pathologic tau formation. The current study investigated the relative density of tau-positive inclusions and neuronal counts exclusively in the DG of the hippocampus across four distinct tauopathies: PiD, CBD, PSP, and AD. The latter was assessed in both aphasic (i.e., PPA) and amnestic (i.e., DAT) clinical phenotypes to determine whether 3R/4R tau pathology of AD in the DG varies with the absence or presence of memory deficits at onset. Neuronal densities were quantified to identify relationships between pathologic burden and patterns of neurodegeneration in the DG. Findings revealed differential patterns of susceptibility of DG cell types (granule cells vs hilar neurons) to tauopathies, offering potential insights into hippocampal function and selective vulnerability of the DG to disease.

## Materials and methods

### Participant characteristics and demographic information

Thirty-two right-handed participants with clinical diagnoses of PPA and an autopsy-confirmed tauopathy (PiD, N = 8; CBD, N = 8; PSP, N = 8; AD, N = 8) as the primary pathologic diagnosis were included in this study. These participants were recruited by the longitudinal NIH-funded PPA Research Program and co-enrolled into the NIA-funded Northwestern University Alzheimer’s Disease Research Center. An additional 5 right-handed participants with clinical diagnoses of DAT and postmortem AD were identified for comparison with the PPA/AD group. All cases had no known genetic mutations. Written informed consent and agreement to enter the brain donation program were obtained from all participants in the study, and the study was approved by the Northwestern University Institutional Review Board and in accordance with the Helsinki Declaration (www.wma.net/en/30publications/10policies/b3/). The diagnosis of PPA was based on the criteria of Mesulam [9, 13] and required a clinical history of progressive language impairment unaccompanied by consequential decline in other cognitive domains within the initial stages of the disease. Of the PPA participants, 18 were assigned a clinical subtype of agrammatic/non-fluent, both with and without motor speech deficits. Five participants were of the logopenic subtype, all of whom except for one had an AD pathologic diagnosis. One PPA-PiD participant was of the semantic subtype. Additional file 1: Table 1 provides clinical and pathologic information on each participant included in this study. Except for cases PPA-AD 6 and 8, all DAT-AD and PPA-AD cases were also included in Gefen et al., 2012 [16]. Cases PPA-AD 6 and 8 were previously included in Ohm et al., 2021 [29]. In the 32 PPA participants, average PMI was 15.5 h and average brain weight was 1,113 g. The mean age at death was 69.75 years for PiD participants, 75 years for CBD participants, 80 years for PSP participants, 72.6 years for PPA-AD, and 81.4 years for DAT-AD. See Table 1 for demographic and participant information at the group level.

**Table 1:** Participant characteristics and demographic information

	PPA-PiD (N = 8)	PPA-CBD (N = 8)	PPA-PSP (N = 8)	PPA-AD (N = 8)	DAT-AD (N = 5)	Overall (N = 37)
Sex (% Female)	50%	62.5%	62.5%	25%	60%	51%
Age at Onset (yrs; mean ± SD)	59 ± 4.9	64.9 ± 4.5	71.3 ± 6.6	62.3 ± 10.1	70.2 ± 9.1	65.1 ± 8.3
Age at Death (yrs; mean ± SD)	69.8 ± 4.3	75 ± 6.6	80 ± 7.4	72.6 ± 9.1	81.4 ± 6.7	75.3 ± 7.9
Disease Duration (yrs; mean ± SD)	10.8 ± 2.5	10.1 ± 3.9	8.8 ± 2.9	10.4 ± 3.3	11.2 ± 4.1	10.2 ± 3.2
PMI (hrs; mean ± SD)	12.9 ± 10.5	15.6 ± 14.2	19.3 ± 20.4	14.3 ± 6.8	21.8 ± 28.2	16.3 ± 15.9
Brain Weight (g; mean ± SD)	1011.6 ± 91.2	1112.1 ± 118.8	1179.6 ± 130.4	1151.7 ± 150.7	1178 ± 147.2	1119 ± 134
Education (yrs; mean ± SD)	15.6 ± 2.4	16.3 ± 2.3	15.5 ± 2.1	16.5 ± 2.6	14.4 ± 2.6	15.8 ± 2.3
ApoE 4 Frequency (%)	14.3%	7.1%	6.3%	25%	50%	18.6%

*SD* standard deviation, *PPA* primary progressive aphasia, *PiD* Pick’s Disease, *CBD* corticobasal degeneration, *PSP* progressive supranuclear palsy, *AD* Alzheimer’s Disease, *DAT* dementia of the Alzheimer’s type, *PMI* postmortem interval, *ApoE *apolipoprotein E

### Neuropathologic evaluation and histological preparation

Following autopsy, the cerebral hemispheres were separated in the midsagittal plane, cut into 3- to 4-cm coronal slabs, fixed in formalin for 2 weeks or 4% paraformaldehyde for 36 h, taken through sucrose gradients (10–40%) for cryoprotection, and stored in 40% sucrose with 0.02% sodium azide at 4 °C. The pathologic diagnosis of FTLD and specification of its variants were rendered by neuropathologists (E.H.B and M.E.F) using the published consensus criteria of the Consortium for FTLD [30]. AD neuropathologic change was assessed using criteria set by Montine et al. and Hyman et al. [31, 32]. All cases carrying an AD pathologic diagnosis were characterized as having high AD neuropathologic change. In addition to a primary tauopathy, a subset of PPA specimens contained comorbid pathologies. These included diffuse neocortical (CBD 6) and limbic Lewy body pathology (CBD 7 and PSP 3), and medial temporal TDP-43 pathology (PSP 1, 2, and 7). TDP-43-immunopositivity was confined to the amygdala and hippocampal complex only. For all cases, samples were taken from the posterior third of the left hippocampus, embedded in paraffin, and cut into 5 μm-thick sections. Sections were stained immunohistochemically with an anti-human phosphorylated tau antibody [AT8 (Ser202, Thr205); mouse monoclonal; Invitrogen MN1020; 1/500] to visualize tau pathology. A subset of 5 PPA-AD cases (PPA-AD 1, 4, 5, 7 and 8) were additionally stained with 3R [RD3 (clone 8E6/C11); mouse monoclonal; Sigma-Aldrich; 1/1000] and 4R tau [ET3; gifted by the late Dr. Peter Davies, Albert Einstein College of Medicine, NY; 1/1000] to reveal the proportion of 3R vs 4R tau isoform. Histological staining was performed using 1.0% cresyl violet Nissl stain to visualize neurons for counting.

### Modified stereological analysis of tau-positive inclusions and neurons

Regions of interest were analyzed at 40–63 × magnification. Modified stereological analysis was carried out according to procedures previously described in detail [33], employing the Fractionator method and the StereoInvestigator software (MicroBrightField). All parameters for analysis were tested and adjusted so that the coefficient of error was < 0.1. Five adjacent sections were used to quantify tau inclusions and three adjacent sections for neurons. For each section, the dentate gyrus was traced by following the outer granule cell layer. The top and bottom 1 µm of each section were set as guard height and dimensions of the counting frame were 100 × 100 µm, regardless of magnification. Granule and hilar neurons were counted separately. Inclusions were assessed in terms of tau-positivity irrespective of morphological subtype and were counted in cells with the morphology of neurons. Stereological counts of inclusions and neurons obtained for all sections per brain area were expressed as mean count per cubic millimeter, based on planimetric calculation of volume by the fractionator software. Mean densities were compared between groups to evaluate quantitative differences in tau inclusion densities and neuron densities across tauopathies. Inclusion-to-neuron ratios were computed (e.g., density of AT8-positive inclusions/density of neurons) to analyze the relationship between presence of inclusions and neurodegeneration (i.e., neuronal loss). The ratio of 3R-to-4R tau was assessed by dividing the stereological density of 3R-positive inclusions by the density of 4R-positive inclusions in granule and hilar cells independently.

### Statistical analysis

Fisher’s Exact test was used to analyze differences in ApoE ε4 allele frequency between AD and FTLD-tau cases for which genotyping was obtained. Brown-Forsythe and Welch ANOVA tests were used to compare tau pathologic burden, neuronal densities, and inclusion-to-neuron ratios across tauopathies in granule, hilar, and total (granule + hilar) cell populations; Benjamini and Hochberg corrections were employed to correct for multiple comparisons. A Kolmogorov–Smirnov (KS) test for equality of distributions was used to confirm normality. Adjusted Welch’s t-tests were employed to compare tau burden between PPA-AD and DAT-AD cases. To compare densities between tau inclusions and neuronal counts of hilar or granule cells within a given tauopathy group, Benjamini-Hochberg-adjusted Students’ t-tests were used. Significance level was set at *p* < 0.05.

## Results

### Clinical findings & demographics

There were no significant differences in postmortem interval, disease duration, and education between PPA pathologic groups (Table 1). Fifty percent of PPA participants were female. PPA-PSP participants had significantly higher age of onset (*p* < 0.01) and age at death (*p* < 0.05) compared to PPA-PiD. The Braak staging of NFTs in both the PPA-AD and DAT-AD groups ranged from V to IV. Fifty percent of PPA-AD participants had at least one ε4 allele, compared to 28%, 14%, and 12.5% in PiD, CBD, and PSP, respectively. PSP was the only group that included participants with an ε2 allele (25%). There was no significant difference in ε4 allele presence between pathologic groups in PPA participants. Participants with AD pathology as their primary pathologic diagnosis (DAT + PPA) showed a significantly higher frequency of ε4 alleles (62%) compared to non-AD tauopathy cases (9%)  (*p* = 0.01). Initial Clinical Dementia Rating (CDR) score was available for 26 PPA participants and ranged from 0 to 0.5.

### Differential susceptibility of dentate gyrus neurons across tauopathies in PPA

**Fig. 1:**
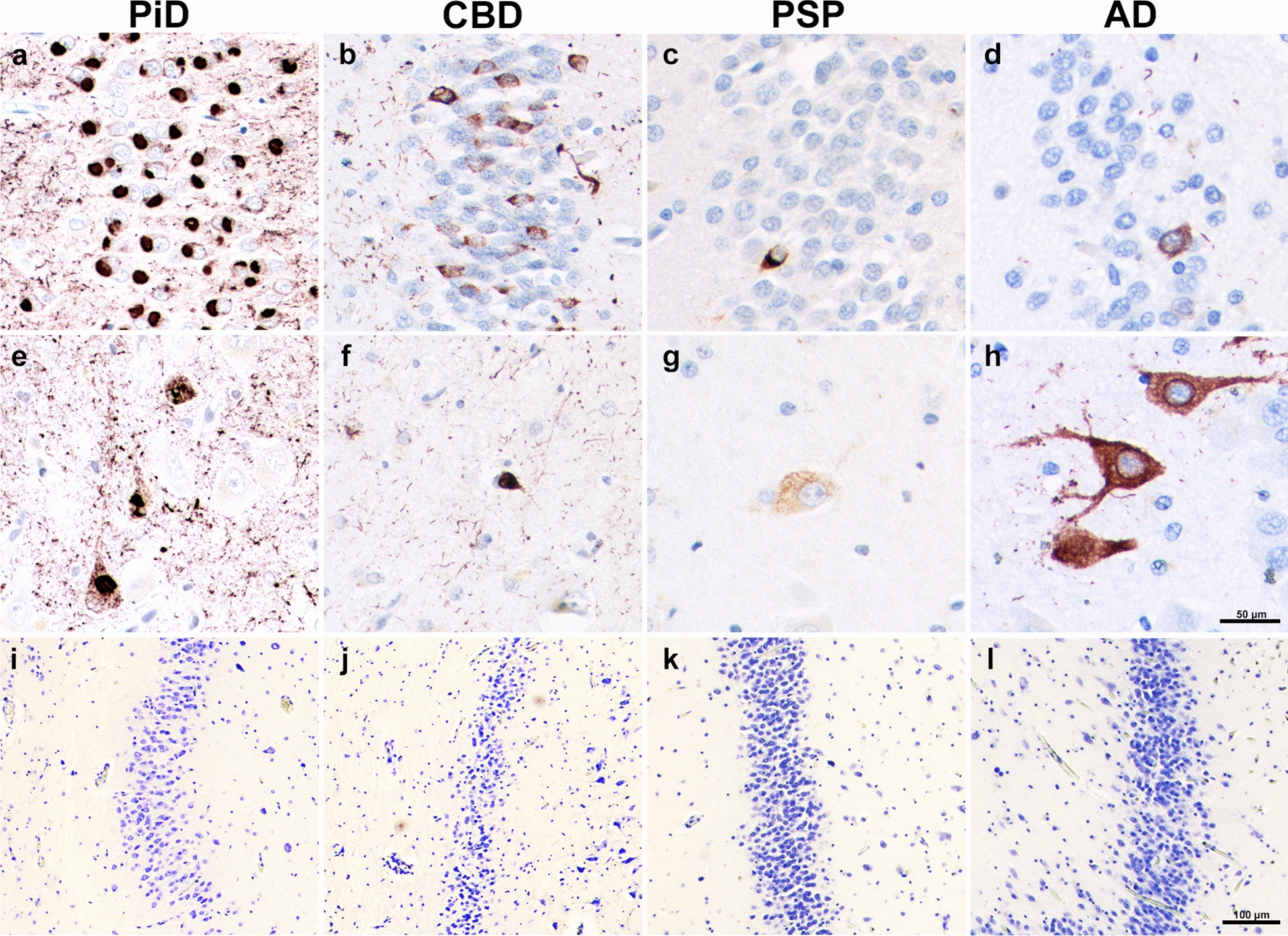
Tau-positive Pathology and Neurons in Aphasic Dementia. (**a–d**) Granule cell tau pathology visualized through AT8 immunohistochemical staining. (**e–h**) Hilar tau pathology revealed through AT8 immunohistochemical staining. (**i–l**) Granule neuronal density revealed through histochemical staining with cresyl violet. (**a, e, i**) was taken from case PiD 7, (**b, f, j**) was taken from CBD 6, (**c, g, k**) was taken from PSP 4, and (**d, h, l**) was taken from PPA-AD 3. PiD images show intense tau neuropathologic burden in both granule (**a**) and hilar (**e**) cells. CBD images show greater neuropathologic inclusions in granule cells (**b**), while PPA-AD has more inclusions in the hilar region (**h**). PSP shows uniformly sparse pathology (**c, g**). While PSP and AD show relatively preserved neuronal densities (**k, l**), there is no significant difference between neuronal densities across tauopathies. Scale bar = 50 µm in **h**, and applies to **a–g**. Scale bar = 100 µm in **l**, and applies to **i–k**

Highest mean densities (inclusions per mm^3^) of DG tau pathology were found in PPA-PiD cases (M = 77,105; SD = 30,642), followed by PPA-CBD (M = 15,611; SD = 15,654), PPA-AD (M = 5,674; SD = 3,549), and lastly PPA-PSP (M = 1,119; SD = 3,549). Total mean DG tau burden in PiD was significantly greater compared to all other groups (*p* < 0.05); PiD densities were greater than CBD, AD, and PSP by order of fivefold, 15-fold, and 70-fold, respectively. We also quantified tau inclusions by cell type, specifically granule and hilar cells, and found that distributions varied greatly between the tauopathy subtypes. In general, granule cell inclusion densities followed the same pattern as overall DG tau densities, with PiD showing significantly more mean granule inclusions compared to all other PPA pathologic groups (*p* < 0.005). PPA-AD cases showed ~ 2X more inclusions in hilar compared to granule cells (*p* < 0.05). In contrast, CBD, and particularly PiD cases contained significantly greater mean density of inclusions in granule cells. CBD cases showed about 9X more inclusions in the granule cells compared to the hilus (*p* < 0.05), and PiD had 15X more granule inclusions (*p* < 0.0001). Despite selective granule cell burden, PiD also had the greatest mean hilar inclusions, reaching significance compared to cases with CBD and PSP (*p* < 0.005). In PSP, granule and hilar cells had equally sparse tau pathologic burden, with levels that were significantly lower than PPA-AD cases (*p* < 0.05). See Fig. 1a–h**.**

### Densities of dentate granule and hilar neurons in PPA due to tauopathy

**Fig. 2:**
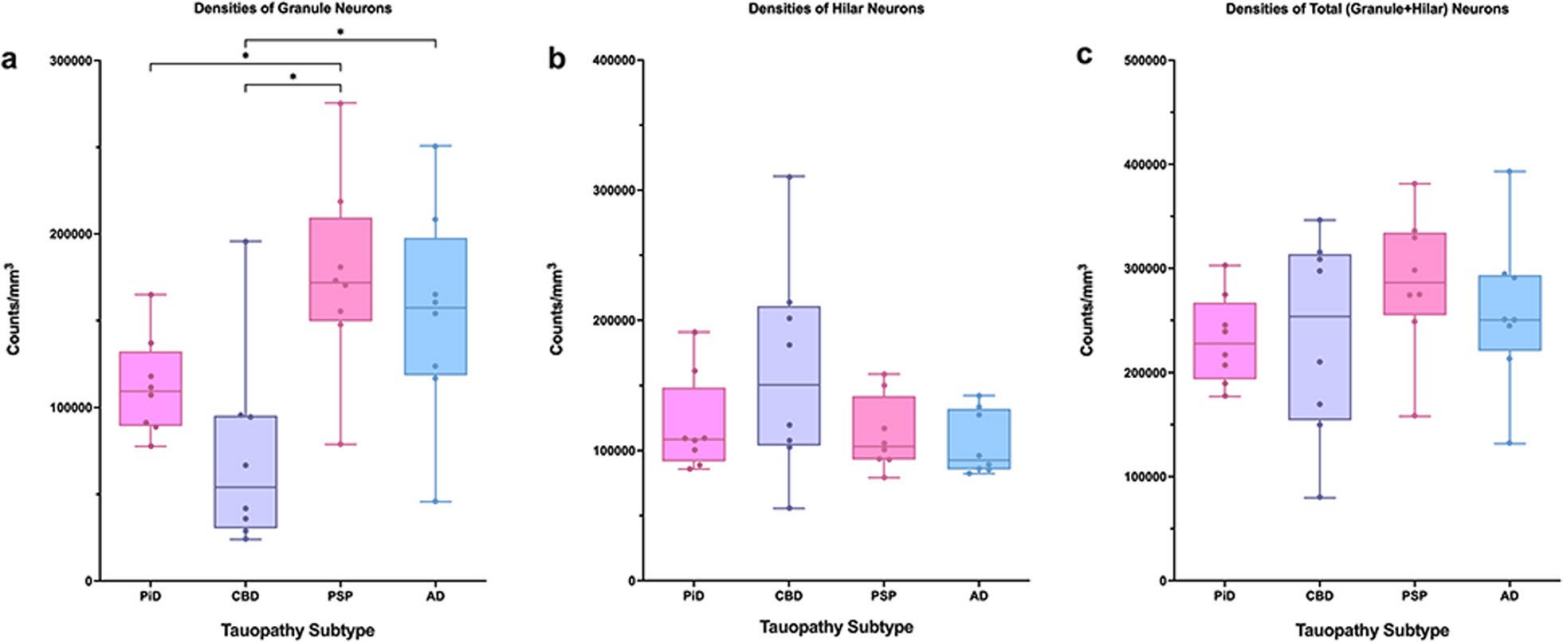
Densities of Dentate Gyrus Neurons in PPA due to Tauopathies. Box and whisker plots show densities of granule (**a**), hilar (**b**), and total (**c**) neurons in the dentate gyrus of PPA-PiD (N = 8), -CBD (N = 8), -PSP (N = 8), and -AD (N = 8) cases. PSP cases showed a significantly higher density of granule neurons compare to PiD and CBD (*p* < 0.05), and AD cases demonstrated a significantly higher density of granule neurons than CBD cases (*p* < 0.05). Total neuronal density was generally equal across all tauopathies. Whiskers indicate minimum and maximum densities, and the box delineates lower and upper quartiles. Each point represents the neuronal density of an individual case. Line within the box represents median

Mean neuronal densities were stereologically quantified in all PPA cases. Qualitatively, we found no meaningful differences in neuronal densities between the small number of cases with comorbid pathology to those without. PiD and CBD had on average fewer overall (granule + hilar) DG neurons compared to PSP and AD cases, with PSP cases containing the most overall neurons (Fig. 1i–l). These overall neuronal densities were not significantly different across tauopathy groups. When granule cells were quantified in isolation, PSP and AD again showed greater mean neuronal densities compared to PiD and CBD cases; the PSP group demonstrated significantly more granule cells compared to CBD and PiD (*p* < 0.05). Interestingly, the PiD group showed greater mean counts of preserved granule cells compared to CBD (M = 112,000 neurons per mm^3^; SD = 28,435 *versus* M = 72,000 neurons per mm^3^; SD = 57,123, respectively), though this difference did not reach significance. CBD cases showed the greatest hilar neuronal density, followed by PiD, PSP, and then AD; these differences also did not reach significance. Relative to each other, the latter three groups showed similar mean hilar neuronal counts, ranging from 105,000 to 120,000 neurons per mm^3^. See Fig. 2**.**

### Relationship between dentate tau inclusions and neurons in PPA due to tauopathy

**Fig. 3:**
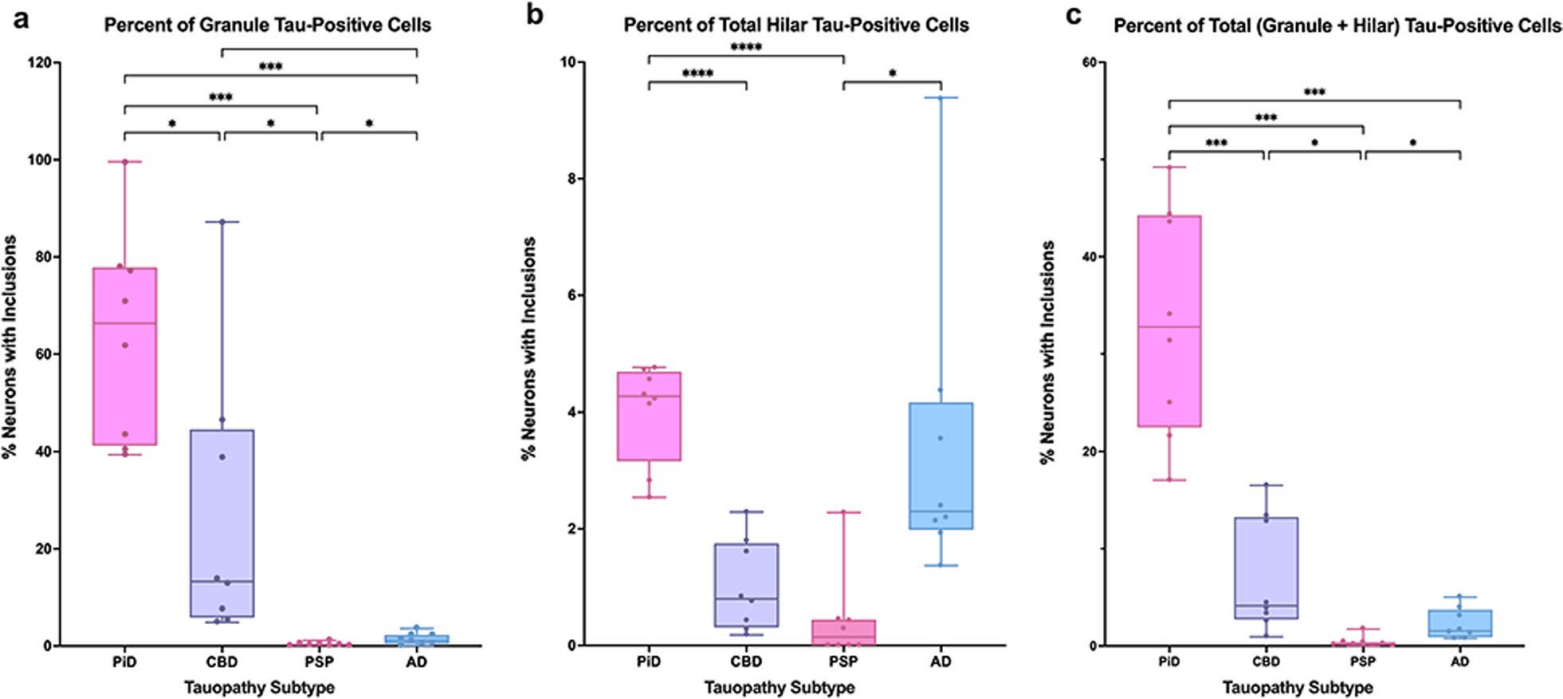
Percent of Dentate Gyrus Neurons affected by Tau-positive Inclusions in PPA. (**a–c)** Box and whisker plot show percent of neurons containing an inclusion in the dentate gyrus of PiD (N = 8), CBD (N = 8), PSP (N = 8), and AD (N = 8) cases, all with a clinical diagnosis of PPA. Percent of neurons containing an inclusion in granule **(a)**, hilar **(b)**, and all **(c)** dentate gyrus neurons was found by calculating an inclusion-to-neuron ratio and converting the quotient into a percent. Patterns of significance in ratio data closely followed those of inclusion data, where PiD showed a higher percent of tau-positive granule and hilar cells than all other groups. CBD showed a slight predilection for granule cell tau-positivity, and AD had a clear preference for tau-positivity in hilar regions. *p < 0.05; **p < 0.01; ***p < 0.001; ****p < 0.0001. Note the large difference in the relative scale of the y-axis across all bar graphs. Whiskers indicate minimum and maximum densities, and the box delineates lower and upper quartiles. Each dot represents an individual case. Line within the box represents median

A ratio of inclusions to neurons was calculated to examine the mean percent of total neurons that contained tau-positive inclusions (Fig. 3). We found that this ratio tracked closely with inclusion density trends, which is consistent with a lack of significant difference between overall (granule + hilar) neuronal densities across tauopathy groups. In PiD cases, ~ 64% of granule cells and 4% of hilar cells contained tau inclusions on average, with 1/3 (~ 33%) of all neurons (granule + hilar) in PiD cases containing a tau inclusion. This burden is striking compared to ~ 7% of all DG neurons affected in CBD, 2% in AD, and 0.4% in PSP.

### Alzheimer’s tau pathology in the dentate gyrus in aphasic (PPA) versus amnestic (DAT) dementia

**Fig. 4:**
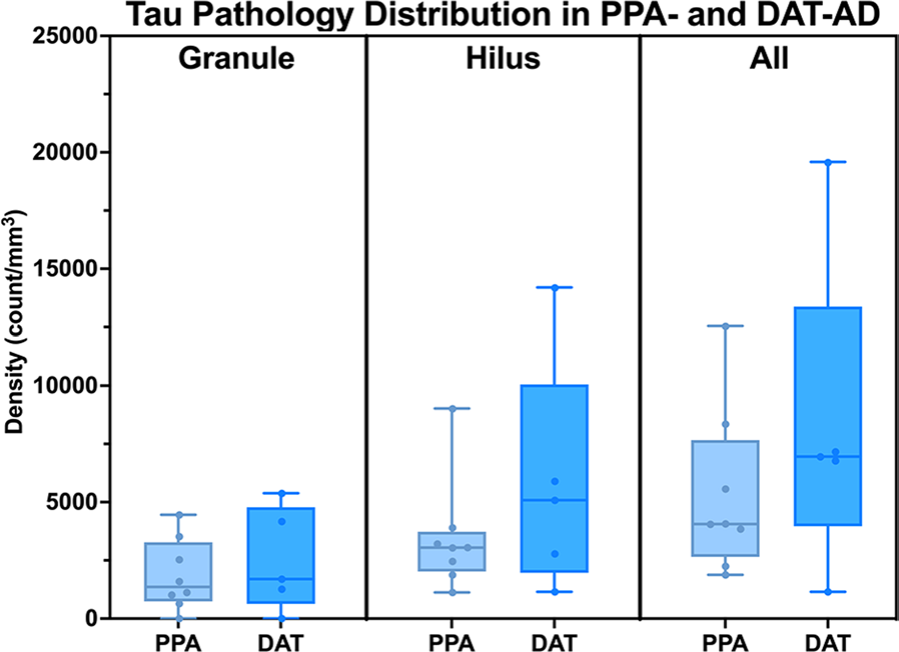
Density of Tau Pathology in Amnestic (PPA) vs Aphasic (DAT) Dementia due to Alzheimer’s Disease. Box and whisker plot show the densities per cubic millimeter of neurofibrillary tau pathology of the AD type in participants diagnosed antemortem with primary progressive aphasia (PPA) (N = 8) or dementia of the Alzheimer’s type (DAT) (N = 5). Total DG counts (granule + hilar) are provided as well as stratified by granule and hilar cell populations. While DAT-AD had slightly more overall and hilar tau pathology, this difference did not reach significance. Whiskers indicate minimum and maximum densities, and the box delineates lower and upper quartiles. Each dot represents an individual PPA-AD or DAT-AD case. Line within the box represents median

**Fig. 5:**
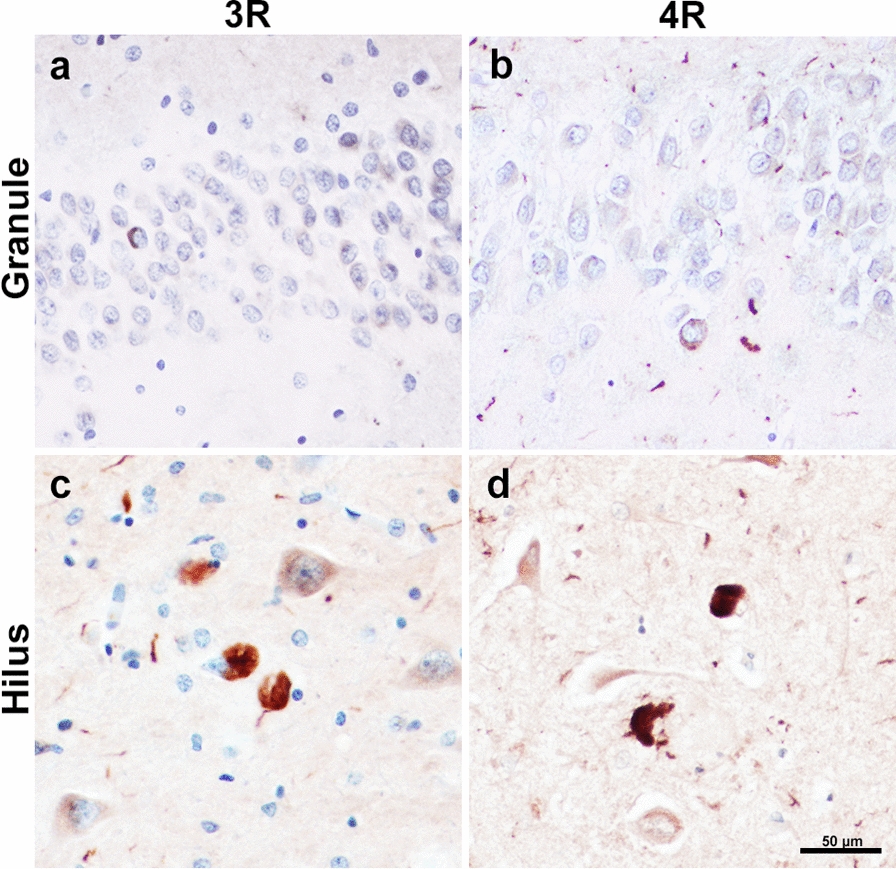
3R- and 4R-tau-positive Inclusions in PPA due to Alzheimer’s Disease. 3R-positive pathology visualized through RD3 immunohistochemical staining in granule (**a**) and hilar cells (**c**); taken from case PPA-AD 7. 4R-positive pathology visualized through ET3 immunohistochemical staining in granule (**b**) and hilar cells (**d**); taken from case PPA-AD 5. Ratio of stereologic densities revealed ~ 1:1 ratio of 3R-to-4R immunopositivity in the granule cells, and ~ 1.5:1 ratio in hilar cells. Differences in 3R and 4R densities were not significantly different. Scale bar = 50 µm in **d**, and applies to **a–c**

To elucidate the relative impact of Alzheimer’s tau pathology on clinical phenotype, we compared the mean density of tau inclusions in PPA-AD cases with stereologic data from five participants who carried an antemortem clinical diagnosis of DAT-AD. Comparatively, the mean tau pathologic burden in DAT-AD cases were similar to that in PPA-AD. We found that DAT-AD cases had total (granule + hilar) 8329 mean inclusions per mm^3^ (SD = 6780), compared to a mean of 5764 total inclusions per mm^3^ in PPA-AD (SD = 3549) in the DG. This difference did not reach significance. Further, when the counts were stratified according to granule *versus* hilar cell type, there was once again no significant difference between the two clinical phenotypes (Fig. 4). In a subset of PPA-AD cases, the ratio of 3R-to-4R inclusion densities in the granule cells was ~ 1:1.1, with a mean 3R density of 1927 inclusions per mm^3^ (SD = 1438) and a mean 4R density of 1724 inclusions per mm^3^ (SD = 1277). Hilar inclusions had a 3R-to-4R ratio of ~ 1.5:1. Mean 3R-positive inclusions in the hilus was 5690 inclusions per mm^3^ (SD = 2708) and mean 4R-positive inclusions in the hilus was 3631 inclusions per mm^3^ (SD = 2339). These differences were not significant (Fig. 5).

## Discussion

In both typical amnestic dementia and normal aging, memory impairments have been correlated with early appearance of Alzheimer’s disease (AD) tau neuropathology in regions of the hippocampal formation. However, the dentate gyrus (DG) of the hippocampus appears to be protected from the formation of the tau-rich neurofibrillary tangles (NFTs) characteristic of AD, despite its key role in memory functioning. In this study, we found that the DG was differentially vulnerable to distinct tauopathies, with a particular affinity to 3R PiD. Within the hippocampal complex, we observed that specificity was dependent on cell type, with certain tauopathies affecting granule or hilar cells to variable degrees. Lastly, compared to cases diagnosed with a typical amnestic dementia syndrome due to AD, we found no significant difference in tau inclusion burden in the DG in cases diagnosed with PPA due to AD.

Molecularly, pathological tau inclusions may contain either three or four microtubule-binding repeat domains (i.e., 3R vs 4R tauopathy) or both, which tend to show differing morphologies as a function of 3R and 4R predominance [23, 24]. Further, DAT-AD, PPA-AD, and FTLD-tauopathies show different vulnerability profiles and temporal patterns of distribution. NFTs in early DAT-AD and aging appear in the paralimbic/limbic ERC and hippocampus for reasons that are not yet fully understood. NFTs in PPA-AD likely first manifest in inferior parietal and superior temporal regions, most commonly leading to word-finding difficulties characteristic of the logopenic variant of PPA [16, 29, 34, 35]. FTLD-tauopathies are most associated with focal cortical neurodegeneration in frontotemporal regions, leading to frontotemporal dementia syndromes such as PPA and behavioral variant frontotemporal dementia (bvFTD) [36]. However, multiple studies have shown abundant 3R-tau-filled Pick bodies aggregated in the perikaryal cytoplasm of dentate granular neurons [23, 26, 27]. Some studies have offered insights into the link between specialized DG granule cells and 3R tau. One investigation found that DG granule cells express 3R but not 4R tau mRNA [25] and others have provided evidence that 3R tau is present in newly generated cells in the DG and may be a marker for adult neurogenesis [37, 38]. On the contrary, the relative paucity of 4R tau inclusions in the DG in PSP and CBD provides insight into the resistance of tau formation in DG granule cells. The *MAPT* H1 haplotype, for example, has been found to be associated with increased risk in developing PSP [39], CBD [40], and AD (only in non-ApoE-4 carriers) [41], suggesting a common genetic link between tauopathies that contain 4-repeat scripts [42]. Still, the mechanism for aberrant 3R versus 4R tau formation in granule cells during the course of disease requires additional and comprehensive study, both in animal models and in the human brain.

This study also examined the presence of AD pathology exclusively in the DG as it occurs in aphasic (PPA) versus amnestic (DAT) dementia syndromes. Examination of tangle burden in these two clinically distinct dementia phenotypes revealed very sparse tangles in the DG with no significant differences between phenotypes. A recent study from Mesulam et al. [28] showed supportive results, where PPA-AD patients demonstrated preserved memory functioning years into the disease course, despite hippocampo-entorhinal AD neuropathology comparable to that of DAT-AD. One of the most striking features of the hippocampus is the serially arranged and highly ordered chain of connections that link cytoarchitecturally distinct zones. The granule cells of the DG receive input from a variety of sources, primarily through dendritic trees in the molecular layer from the entorhinal cortex. In turn, DG granule cells send a dense, zinc-rich ‘mossy fiber’ bundle that synapses on dendritic spines of neurons in the CA3 region. These neurons then send collaterals of their projecting axons, also known as ‘Schaffer collaterals,’ to CA3 as well as to CA2 and CA1 pyramidal cells [43, 44]. In the *early* topographic hierarchy of NFT accumulation in DAT-AD, it has been shown that the granule cells of the DG are relatively spared from infiltration [8, 45] and subsequent neuronal loss [46]. In later stages there is evidence of sparse dentate NFTs [47], and our findings are concordant with this pattern of late vulnerability. Multiple studies have found the ratio of tau isoforms in NFTs to be 1:1 [25, 48–50] in DAT-AD. This study is the first to show a ~ 1:1 ratio of tau isoforms in the granule cells of the DG in PPA-AD cases, highlighting molecular similarities between granule tau expression in the aphasic versus amnestic phenotype.

The molecular determinants of selective vulnerability of the DG to the 3R tau is particularly puzzling given the preservation of memory functions in PPA cases investigated. One possibility is that while Pick bodies form in the DG in early disease [27], the accumulation of pathology in the DG is slower than that in cortical areas, leading to preserved memory until later disease stages. Another possibility is that the formation of a Pick body is a productive disease response that does not progress to cell death. In PPA cases with the type-C variant of FTLD-TDP, we found that neurons accumulate abnormal cytoplasmic TDP-43 but to an extent that it does not necessarily lead to neuronal degeneration [51]. The Pick body may show an affinity to the granule cell but similarly may not be cytotoxic. Lastly, it is possible that the DG is functionally important to encoding-based memory but not necessarily long-term retentive storage [52–54]. Indeed, electrophysiologic experiments have shown that the ERC can excite CA3 and CA1 neurons without the excitation of DG granule cells [55]. Further, studies have found that the expression of the tetracycline transactivator (TTA) in some mouse strains results in severe, focal DG degeneration and atrophy, yet these mice do not show memory deficits on behavioral testing [56]. This finding, along with the presence of DG pathology in non-amnestic dementias, challenges the supposed importance of a fully intact DG in memory. A critical next step would be to investigate the functional and pathologic vulnerabilities of all transsynaptic nodes along the hippocampal circuit affected by hierarchical disease spread that exist beyond the DG.

Multiple clinicopathologic studies have suggested that the clinical dementia phenotype is more tightly linked to the location of the pathology rather than its molecular determinants [10, 18, 19, 21, 22]. While location appears to be the meaningful driver of clinical phenotype, findings from this study bring forth an exciting nuance in that it considers the selective vulnerabilities of specific cell groups to distinct tau species within a single anatomic region. Limitations to this study include small cohort size, lack of morphologic inclusion specificity, and modifications to stereological methodology given tissue preparation. Future studies will investigate the interaction between tau pathology, neuronal loss and size, and synaptic and axonal integrity along well-known neurobehavioral circuits to better discern the complex factors that contribute to functional and structural damage in dementia.

## Supplementary Information

**Additional file 1**. Individual characteristics of all cases.

## Data Availability

The datasets used and/or analyzed during the current study are available from the corresponding author on reasonable request.

## Abbreviations

DG: Dentate gyrus
AD: Alzheimer’s disease
NFT: Neurofibrillary tangle
DAT: Dementia of the Alzheimer’s type
PPA: Primary progressive aphasia
FTLD-tau: Frontotemporal lobar degeneration of the tau form
PiD: Pick’s disease
CBD: Corticobasal degeneration
PSP: Progressive supranuclear palsy
