# Supplementary material for: Differential vulnerability of the dentate gyrus to tauopathies in dementias
Source: Acta Neuropathol Commun. 2023 Jan 3;11:1. doi: 10.1186/s40478-022-01485-7 (PMC9811688; doi:10.1186/s40478-022-01485-7)
Supplement: Supplementary file 1 — Additional file 1. Individual characteristics of all cases. [file 40478_2022_1485_MOESM1_ESM.pdf]

Supplementary Data

Table 1. Characteristics

	Sex	Age at Death (yrs)	PMI (hrs)	Brain Weight (g)	Age of Onset	Disease Duration (yrs)	Education (yrs)	ApoE	Clinical Dx	Tauopathy
PiD 1	M	75	27	1125	66	9	14	UNK	PPA (Mixed)	PiD
PiD 2	M	65	UNK	1055	53	12	14	3,3	PPA-G	PiD
PiD 3	F	76	3.5	965	64	12	16	3,4	PPA-G	PiD
PiD 4	M	70	5	1018	56	14	20	3,3	PPA-G	PiD
PiD 5	F	68	14	1010	60	8	16	3,3	PPA-G	PiD
PiD 6	F	64	28	990	53	11	16	3,4	PPA-S	PiD
PiD 7	M	69	5	1100	62	7	12	3,3	PPA (Severe)	PiD
PiD 8	F	71	8	830	58	13	17	3,3	PPA (Unsub)	PiD
CBD 1	F	72	16	980	67	5	14	UNK	PPA-G	CBD
CBD 2	F	74	5	1168	66	8	14	3,3	PPA-G	CBD
CBD 3	F	71	9	990	62	9	18	3,3	PPA-Lˆ	CBD
CBD 4	M	83	8	1080	65	18	20	3,4	PPA-G	CBD
CBD 5	M	75	46	1349	65	10	16	3,3	PPA-G	CBD
CBD 6	F	69	UNK	1050	60	9	18	3,3	PPA-G	CBD
CBD 7	M	69	18	1160	60	9	16	3,3	PPA-G	CBD
CBD 8	F	87	7	1120	74	13	14	3,3	PPA (Mixed)	CBD
PSP 1	F	82	16	1280	77	5	14	3,4	PPA (Severe)	PSP
PSP 2	F	82	9	1060	70	12	16	3,3	PPA-G	PSP
PSP 3	F	66	17	1120	62	4	14	3,3	PPA-Gsp	PSP
PSP 4	F	88	7	970	78	10	14	3,3	PPA-G	PSP
PSP 5	M	73	25	1262	63	10	14	2,2	PPA-G	PSP

PSP 6	M	78	6	1100*	68	10	16	3,3	PPA-Gsp	PSP
PSP 7	M	87	7	1315	79	8	20	2,3	PPA-G	PSP
PSP 8	F	84	67	1250	73	11	16	3,3	PPA-G	PSP
PPA-AD 1	F	77	18	1250	71	6	16	3,3	PPA-L	AD
PPA-AD 2	M	71	24	1250	59	12	15	3,4	PPA (Mixed)	AD
PPA-AD 3	M	87	6	1100	80	7	16	3,4	PPA-L	AD
PPA-AD 4	M	81	6	1110*	68	13	20	3,3	PPA-G	AD
PPA-AD 5	M	75	8	1030*	62	13	19	3,4	PPA-L	AD
PPA-AD 6	M	63	14	1120	55	8	13	3,3	PPA (Mixed)	AD
PPA-AD 7	F	66	19	890	51	15	19	3,3	PPA-L	AD
PPA-AD 8	M	61	19	1300	52	9	14	3,4	PPA (Severe)	AD
DAT-AD 1	M	80	20	1390	74	6	14	3,4	DAT	AD
DAT-AD 2	M	71	6	1210	54	17	12	4,4	DAT	AD
DAT-AD 3	F	89	71	980	76	13	18	3,4	DAT	AD
DAT-AD 4	F	82	7	1170	73	9	16	3,3	DAT	AD
DAT-AD 5	F	85	5	1140	74	11	12	3,4	DAT	AD

M=male; F=female; *=fixed; PMI=postmortem interval; UNK=unknown; ApoE=apolipoprotein E; PPA=primary progressive aphasia; DAT=dementia of the Alzheimer's type; G=agrammatic; Gsp=agrammatic with motor speech deficits; L=logopenic; S=semantic; Lˆ=Logopenic with mild repetition problems; (Mixed)=patient displayed a mixed subtype; (Severe)=patient was too severe to subtype accurately; (Unsub)=patient was not subtyped; PiD=Pick's Disease; CBD=corticobasal degeneration; PSP=progressive supranuclear palsy; AD=Alzheimer's Disease
